# Remedial Measures in Obstruction of the Common Bile Duct*Abstract of paper read before the American Surgical Association, April 20, 1898, at the New Orleans meeting.

**Published:** 1898-04

**Authors:** J. McFadden Gaston

**Affiliations:** Atlanta, Ga.


### Southern Medical Record.

A MONTHLY JOURNAL OF MEDICINE AND SURGERY.

VOL. XXVIII. ATLANTA, GA., APRIL, 1898. NO. 4.

### Original Articles.

### *REMEDIAL MEASURES IN OBSTRUCTION OF THE COMMON BILE DUCT.

BY J. McFADDEN GASTON, M. D., ATLANTA, GA.

The cases of jaundice which result from the temporary closure or constriction of the common bile duct are usually left to the treatment of the physician and yet are the precursors of derangements which require surgical interference and hence come within the sphere of this paper. They commence with funcional disturbance, but terminate in organic derangements.

Murphy lays great distress upon the relief of jaundice by pilocarpine. The reliance of the greater number of practitioners for relief in this class of cases has been and continues to be upon the phosphate of soda. But there have been more good results claimed from the employment of olive oil, internally, than from any other remedy of this class. After exhausting medico-chirurgical means of treatment for biliary complications, without arresting obstruction of the common bile duct, the surgeon is confronted with conditions calling for operative interference. There may exist a stenosis or stricture of the walls of the duct, without occlusion of the canal, dependent upon inflammation, or there may be a partial obstruction of the lumen of the duct from the presence of a gall-stone which permits the bile to pass around it to a limited extent. In some cases, Fenger has demonstrated that a gall-stone may be so located as to form a valvular closure. Though an outlet may be artificially provided for the bile by connecting an opening in the gall-bladder with one in the abdominal wall, there is not always relief to the cholæmia, and

---

* Abstract of paper read before the American Surgical Association, April 20, 1898, at the New Orleans meeting.

its effects upon the general health of the patient continue even to a fatal termination. In a case presenting no enlargement of the liver the incision should be made on a line an inch and a half below the border of the costal cartilages, and should be about three inches in length.

I have elsewhere given an unfavorable opinion in regard to exploratory puncture, and do not consider it entitled to recognition as a means of diagnosis or measure of treatment. The various measures recognized for the relief of obstruction of the common duct by gall-stones are breaking up of the calculi by the needle, crushing by padded forceps, forcing them into the gall-bladder or duodenum, catheterization, and excision through the walls of the duct. In some cases it is impracticable to attach the gall-bladder to the parietal opening or to suture the incision in the wall of the duct when a stone is extracted, and thus it becomes necessary to provide for drainage by packing with gauze, around the field of operation, and leave the ends of the strips extending out of the wound.

In doing operation upon three white women for obstruction of the common bile duct by forming a fistulous opening for the bile externally, the cholæmia persisted in an aggravated form until death supervened in each case. In some conditions of temporary impediment to the flow of bile through the duct into the duodenum, the attachment of the incised gall-bladder to the parietal opening has relieved the obstruction and been followed by the restoration of the bile. It is in order to state that only in complete occlusion of the duct, which has proved intractable to other procedures, has an artificial opening from the gall-bladder or the common bile duct into the alimentary canal been thought proper.

My experiments upon dogs demonstrated the feasibility of this fistulous communication of the gall-bladder with the bowels, and it is a matter of minor importance by what process it is accomplished.

The statistics in the past show that the various modes of procedure are comparatively free from danger and are attended with a marked degree of success, so that we may reasonably expect cholecystenterostomy to be recognized as a safe and efficacious recourse in the future annals of surgery.

From the foregoing considerations, we make the following inferences:

1. That obstruction of the common bile duct may result to a greater or less extent from catarrhal inflammation or from gall-stones.

2. The ordinary accompaniment of any obstruction of the common bile duct is jaundice and the absence of the usual color in the fecal evacuations.

3. In the lesser developments of jaundice, medication is to be relied on for relief, but in the more accentuated and persistent forms operative measures are always requisite.

4. An exploratory operation by incision diagonally beneath the right costal cartilages enables the operator to determine upon the nature of the impediment and the best means of relief.

5. In the various cases we may resort to catheterization, needling, crushing, traction, incision of duct or gall-bladder, with attachment of either to the parietes or intestine.

6. That complete occlusion of the common duct can not be remedied by the outlet of bile through a parietal opening, but calls for a communication of the gall-bladder or the duct with the intestinal canal.

7. The duodenum affords most favorable conditions for this connection, a coil of small intestine comes next in its advantage, and the colon presents least benefits, as it fails to obtain the good effects of the bile for intestinal digestion.

8. The anastomosis of the biliary system with the alimentary canal may be accomplished by different processes, and the selection of the mode best adapted to the case may be left to the choice of the operator.

9. Permanent occlusion of the common duct associated with a patulous cystic duct is the only condition in which cholecystenterostomy is indicated, and for this condition of things it may be considered a radical mode of relief.

10. While a fistulous communication of the gall-bladder or common duct with the alimentary canal is a safe and efficacious mode of relief for all ordinary results of occlusion, it does not afford exemption from the consequences of malignant growth, and hence this kind of obstruction is not included in this paper.

---

A young physician, practicing in a city in Ulster County, New York, recently brought suit against a former patient, a young lady, from whom he was unable to collect a bill of sixty dollars for professional services. The defendant's attorney contested the bill, which he characterized as exorbitant. He said that many of the alleged professional calls were of a social nature. The plaintiff would call at times when he wasn't expected and keep the defendant and her sister from their household duties. In view of the annoyance and loss of time the young lady alleges that she suffered through these visits, she puts in a counter claim of one hundred dollars. The case will be tried before a jury.—*Med. Record.*

